# Influence of respiratory mode on the thermal tolerance of intertidal limpets

**DOI:** 10.1371/journal.pone.0203555

**Published:** 2018-09-05

**Authors:** Sebbi L. Kankondi, Christopher D. McQuaid, Morgana Tagliarolo

**Affiliations:** 1 Department of Zoology and Entomology, Rhodes University, Grahamstown, Eastern Cape, South Africa; 2 Ifremer, UMSR LEEISA (CNRS, UG, Ifremer), Cayenne, France; University of Hong Kong, HONG KONG

## Abstract

Predicting ecological responses to climate change requires an understanding of the mechanisms that influence species’ tolerances to temperature. Based on the idea that air and water breathing animals are differentially suited to life in either medium due to differences in their respiratory morphology, we examined the possibility that the thermal tolerances of co-existing intertidal pulmonate and patellogastropod limpets may differ in different breathing media. We tested this by determining each species’ median lethal temperature (LT_50_) and cardiac Arrhenius breakpoint temperature (ABT) as measures of upper thermal tolerance limits, in air and water. Although all these species can survive in air and water, we hypothesised that the pulmonate limpets, *Siphonaria capensis* and *S*. *serrata*, would have higher thermal limits than the patellogastropod limpets, *Cellana capensis* and *Scutellastra granularis*, in air and *vice versa* in water. The results did not support our hypotheses, since *C*. *capensis* had similar thermal tolerance limits to the pulmonate limpets in air and the pulmonate limpets had thermal tolerance limits similar to or higher than *S*. *granularis* in water. Thus, considering pulmonate and patellid limpets as groups, we found no differences in their collective upper thermal tolerance limits in either medium. We conclude that differences between these two limpet groups in their respiratory morphology do not influence thermal tolerance, but that tolerances are species-specific.

## Introduction

Climate change is a statistically significant change in the long-term state of the global climate, caused by a combination of natural and external anthropogenic activity [1]. One of the most important consequences of climate change is the perceived change in environmental temperatures, which are likely to have numerous consequences for ecosystem level processes [2–6]. It has, therefore, become important to improve our understanding of the impact that climate change may have on individual organisms and thus overall ecosystems [7]. The rocky shore and its inhabitants have frequently been used to examine the ecological effects that global changes in temperature may have [8–11] because of the extreme thermal conditions normally experienced across the shore [12–16].

Intertidal limpets are ecologically important due to their activity as grazers and their interactions with other intertidal organisms [17], while their ability to tolerate the many environmental challenges of the intertidal zone has made them important physiological study subjects [18–21]. Like other intertidal animals, limpets contend with continuously alternating breathing media as the tide ebbs and floods. Linked to this is the evolution of different respiratory structures, for example, the pallial gills of patellogastropod limpets and, the mantle cavity lungs and secondary gills of pulmonate limpets [17, 22, 23]. Structural differences between the respiratory anatomies of these two groups’ can influence their ecology [24–26]. While the pallial gills can be used during aerial respiration, they are primarily suited to aquatic respiration and the pulmonate limpets’ secondary gills are not as adept as pallial gills at aiding aquatic respiration [19, 25, 27]. Therefore, while most patellogastropod limpets are suited to an aquatic lifestyle the pulmonate lung is an advantage to life in air [19, 25, 28, 29]. In fact, Marshall and McQuaid [26] found that the pulmonate system may promote a higher thermal tolerance than that of patellogastropods in air. On this basis, we hypothesized that respiratory morphology influences the metabolism and therefore, thermal tolerance of organisms.

Several measures of thermal tolerance have previously been used to help gain an understanding of how organisms respond to thermal variation in their immediate environment. These include whole organism lethal limit measures, which involve determining an organisms’ critical temperatures and LT_50_ values [30–33] and sub-lethal limit measures such as the detection of heat shock proteins and the measurement of cardiac ABTs [34–37]. In this paper, the response to temperature variation of the patellogastropods, *Scutellastra granularis* and *Cellana capensis*, and the pulmonates, *Siphonaria capensis* and *S*. *serrata*, was compared. The upper thermal limits were estimated by measuring LT_50_ values as a direct measure of mortality rates and the ABT for heart rate under increasing temperature [17, 18]. These species were chosen as model organisms because of their overlapping geographical (S1 Fig) and vertical distributions (S2 Fig) across the shore [38–42]. While both pulmonate species are more common in intertidal zones above the low mid-shore, the patellids are ubiquitous from the subtidal fringe to the high mid-shore [38, 39].

Given the differences in their respiratory anatomy, we postulated that these two groups of limpets would exhibit different thermal tolerances in air and water. Recognising that neither group is exclusively air or water breathing, we hypothesised that: 1. the pulmonate limpets would have higher thermal limits than the patellogastropod limpets in air, and *vice versa* in water; 2. The thermal limits of pulmonate limpets would be greater in air than water, while patellogastropod limpets would show the reverse, with higher thermal limits in water than air.

## Materials and methods

### Ethics statement

Only invertebrate marine molluscs (limpets) were used. All work was conducted under the research permits (RES2014/12 and RES2015/04) for collection and practical experiments issued by the Department of Agriculture, forestry and fishery of the Republic of South Africa.

### Sample sites and collection

A total of 400 individuals of each species were collected from high mid-shore rocks on the south-east coast of South Africa at Kenton-on-Sea (33.68° S, 26.67° E), Port Alfred (33.59° S, 26.89° E) and Cintsa West (32.82° S, 28.12° E), during low tide in austral winter (June–July) in 2014 and 2015. Similar sized individuals (20–30 mm) of each species were collected during low tide by quickly sliding a coarse scalpel between the muscular foot and the substratum. Any limpets not detached at the first attempt were left, to avoid using animals that may have been injured. Specimens were transported to the laboratory within 3 hours in small containers, moistened with sea water and kept inside an insulated box. In the laboratory, the specimens were housed in a 20L glass tank filled with 5L of aerated seawater at 22°C for a minimum of 24h and a maximum of 48h before use. Before experimentation, limpets were submerged in 500mL containers filled with constantly aerated seawater for 1 hour to ensure they were fully hydrated.

### Determining the median lethal temperature (LT_50_)

Thermal limits were determined first by finding the LT_50_ values, using a protocol based on Clarke *et al*. [43]. LT_50_ measurements were carried out on 150 individuals per species in each medium, over three trials (50 individuals/trial) to generate a mean LT_50_ value. During the experiment, limpets were housed in 500mL containers (10 individuals/container) filled with natural aerated seawater to simulate aquatic conditions or dampened with seawater to simulate aerial exposure [44]. Temperatures within the containers were controlled by submerging them in a Grant programmable water bath (GP 200, Grant, Germany). A Fluke 54II thermometer (Fluke cooperation, USA) fitted with a T-type thermocouple (Fluke cooperation and Cromega) was used to measure temperature at the bottom of the containers, which were recorded with a PowerLab recording system.

A wide range of heating rates (10°C/min—1°C/3.5 days) have been used to determine thermal limits in past studies [45]. Similar studies on various intertidal organisms, including limpets, have generally used heating rates between 0.1°C.min^-1^ and 0.3°C.min^-1^ [35, 36, 46, 47]. In this study, temperature was programmed to increase at 0.2–0.4°C.min^-1^ for the LT_50_ measurements (S1 Table) using a water bath, following the ramping protocol described in S3 Fig.

To avoid influencing the results through repeated thermal shock [48, 49] a new batch of specimens was used for each temperature interval. After each test run, limpet mortality was determined after a 24-hour recovery period in the holding tank (S3 Fig) and limpet shell lengths were measured to the nearest 0.02mm using Vernier callipers. Mortality was assessed by probing the limpets for tactile responsiveness using a blunt probe. Limpets were classified as dead if they showed no response to having the foot muscle or the edge of the mantle probed.

### Heart rate measurements

Heart rate measurements were carried out on 20 individuals per species in each medium, housed in a total of 56 500mL containers with 3 individuals/container in 48 of the containers, and 2 individuals/container in the rest. To simulate aquatic conditions, the containers were filled with natural seawater aerated using air stones. To determine thermal limits in air, the containers were dampened with seawater to maintain relatively high levels of humidity during heat exposure [44].

Heart rate was recorded using non-invasive plethysmography [50] by attaching optoelectronic (infrared) sensors (Vishay semiconductors, V69 CNY70 732/735, Germany) to the shells of each limpet near the heart using Pattex super glue (Henkel (Pty) Ltd, South Africa). These sensors produced signals which were amplified by a custom-built preamplifier, after which Triangular-Bartlett smoothing was used to produce an additional smooth trace on a separate channel. The signal was then filtered before being recorded as beats per minute on a computerised recording system (PowerLab/4SP and 430, Chart version 5 and 7, ADInstruments, Australia). The amplitude ranged between 40 and 100mV at a sampling rate of 40Hz. The specimens were exposed to a temperature increase of 30°C from 20–50°C at a rate of 0.25°C.min^-1^, using the Grant programmable water bath heating system whilst simultaneously recording heart rate. Prior to heat exposure, the animals were allowed to settle at 20°C in the water bath for 30 minutes. Limpet mortality and shell lengths were determined after each treatment, as described for the LT_50_ measurements.

### Data and statistical analyses

One *caveat* here is that individuals in the same container during pre-treatment could be considered to be pseudoreplicates. However, species were interspersed during pre-treatments and monitoring of the water bath temperature showed no significant or systematic variation. Similarly, there was no evidence that the presence of conspecifics influenced individual thermal physiology.

The LT_50_ values for each trial were generated from limpet mortality at each temperature interval using probit analysis [51, 52]. Thereafter, mean LT_50_ values were compared between media and respiratory modes using a nested ANOVA with species nested in respiratory mode.

Cardiac thermal response curves were plotted and checked manually to control for artefacts and anomalous trends, for example due to movement of the animals. This meant that the final ABT and heart rate analyses were carried out on fewer than 40 individuals from each species (S2 Table). The temperature ranges used to determine ABT were 25–45°C for all species in air (except *Scutellastra granularis*; 25–40°C) and 25–40°C for all species in water (except *C*. *capensis*; 25–45°C). Outside of these temperature ranges the data points were distributed haphazardly.

Arrhenius plots were then generated from these thermal response curves using Eq 1 to analyse the effect of temperature on heart rate:

$$L_{n}(HR)=L_{n}a-\frac{E_{a}}{R}\times \frac{1}{T}$$ (1)

In Eq 1, *HR* represents the heart rate (bpm), *a* is the normalization constant, *E*_*a*_ is the activation energy (J.mol^-1^), *R* is the ideal gas constant (J.K^-1^.mol^-1^) and *T* is the absolute temperature (K). Piecewise linear regression was used to calculate the breakpoints using transformed heart rate (*L*_*n*_ (*HR*)) and temperature (1/*T*) data. These breakpoints were then converted back into degrees Celsius, to present them as ABTs (°C) before conducting further analyses. The ABT values were then compared using a nested ANOVA as described for the LT_50_ values above.

Once the ABT values were determined, the increase in heart rate as a function of temperature was compared among treatments, by considering only the data points below the ABTs. The slopes (*E*_*a*_/*R*) from the resultant, linear Arrhenius plots were compared using a nested ANOVA as described for the LT_50_ and ABT analyses above.

Tukey HSD analyses were used *post hoc* to determine where significant differences lay for all the ANOVA models.All statistical analyses were performed with Statistica 13.

## Results

### Differences in thermal limits between species and/or media

#### LT_50_

**Fig 1:**
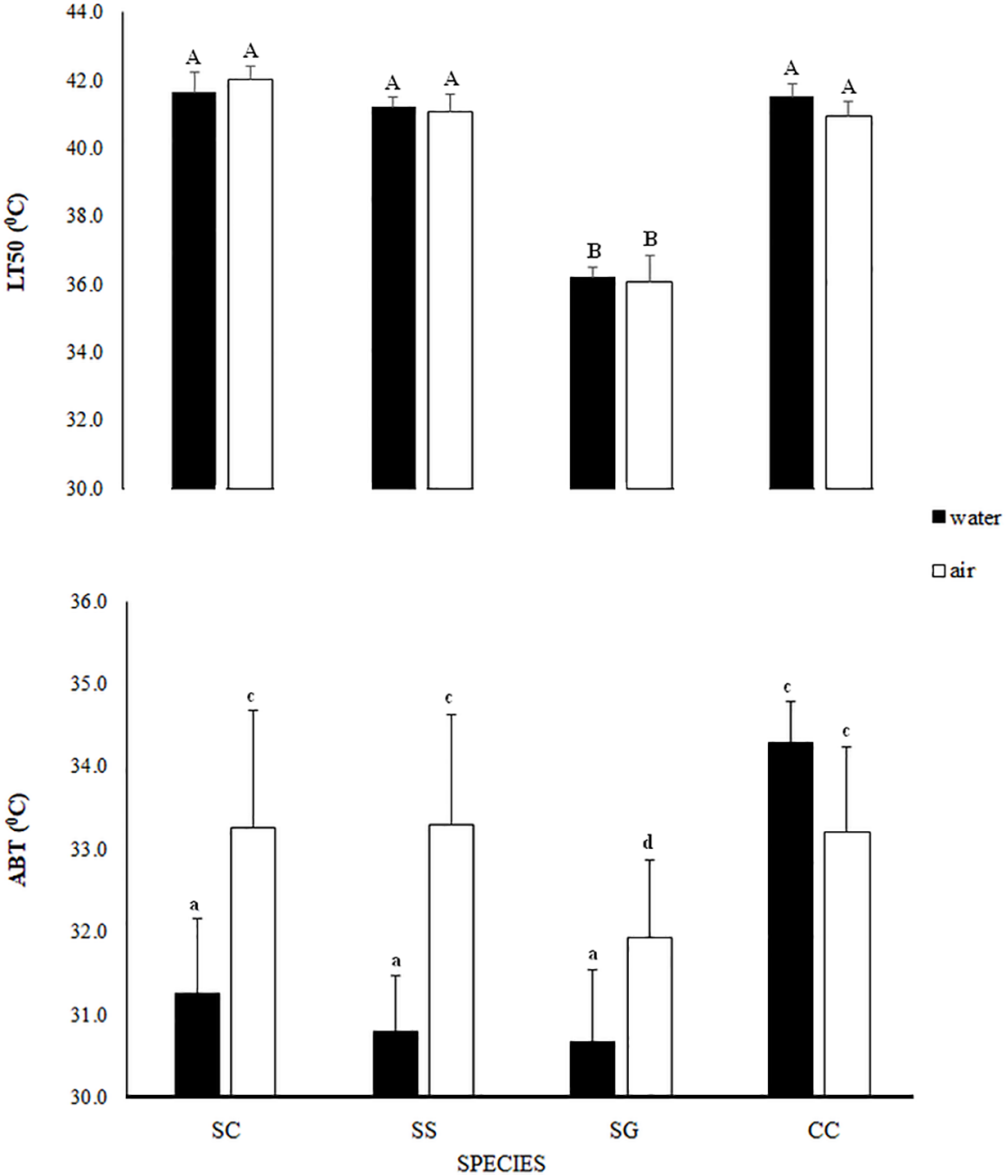
Comparisons of mean (+ S.D) LT_50_ (°C) and ABT (°C) values made among the limpet species in both media, and between media for each species. **Limpet species and corresponding respiratory mode are listed as: SC–*Siphonaria capensis*, Lungs; SG–*Scutellastra granularis*, Gills; CC–*Cellana capensis*, Gills; SS–*Siphonaria serrata*, Lungs.** Homogenous groups are shown in upper case for LT_50_ and lower case for ABT.

The nested ANOVA comparing LT_50_ values (Fig 1; S3 Table), indicated significant effects of Respiratory Mode and of Species nested in Respiratory Mode (p < 0.0001 in both cases), with no effect of Medium or its interaction with Respiratory Mode. In both media, *Scutellastra granularis* had significantly lower values than the other three species, whose LT_50_ values were the same, and there were no significant differences between LT_50_ values in air and water for any species.

The significant effect of Respiratory Mode was not expected on the basis of the raw data and presumably reflects the low LT_50_ of *Scutellastra granularis*, which decreases the mean value for the patellids. This effect is probably exacerbated by the small sample size (n = 3) used in the LT_50_ analysis, particularly compared to the ABT (n ~ 10) analysis.

#### ABT

The nested ANOVA of ABT data (S4 Table) indicated significant effects of Medium, Species nested in Respiratory Mode and the interaction between Medium and Respiratory Mode (p < 0.0001 in all cases). Other than *C*. *capensis*, the limpets had significantly higher ABT values in air than water (Fig 1). Although the effect of Respiratory mode was non-significant in the nested Analysis (p = 0.34), there was a significant effect of Species. *Scutellastra granularis* exhibited a significantly lower ABT in air than the other species, while *C*. *capensis* had a significantly higher ABT in water than the others (p < 0.05 in both cases).

### Relationship between heart rate and temperature

**Fig 2:**
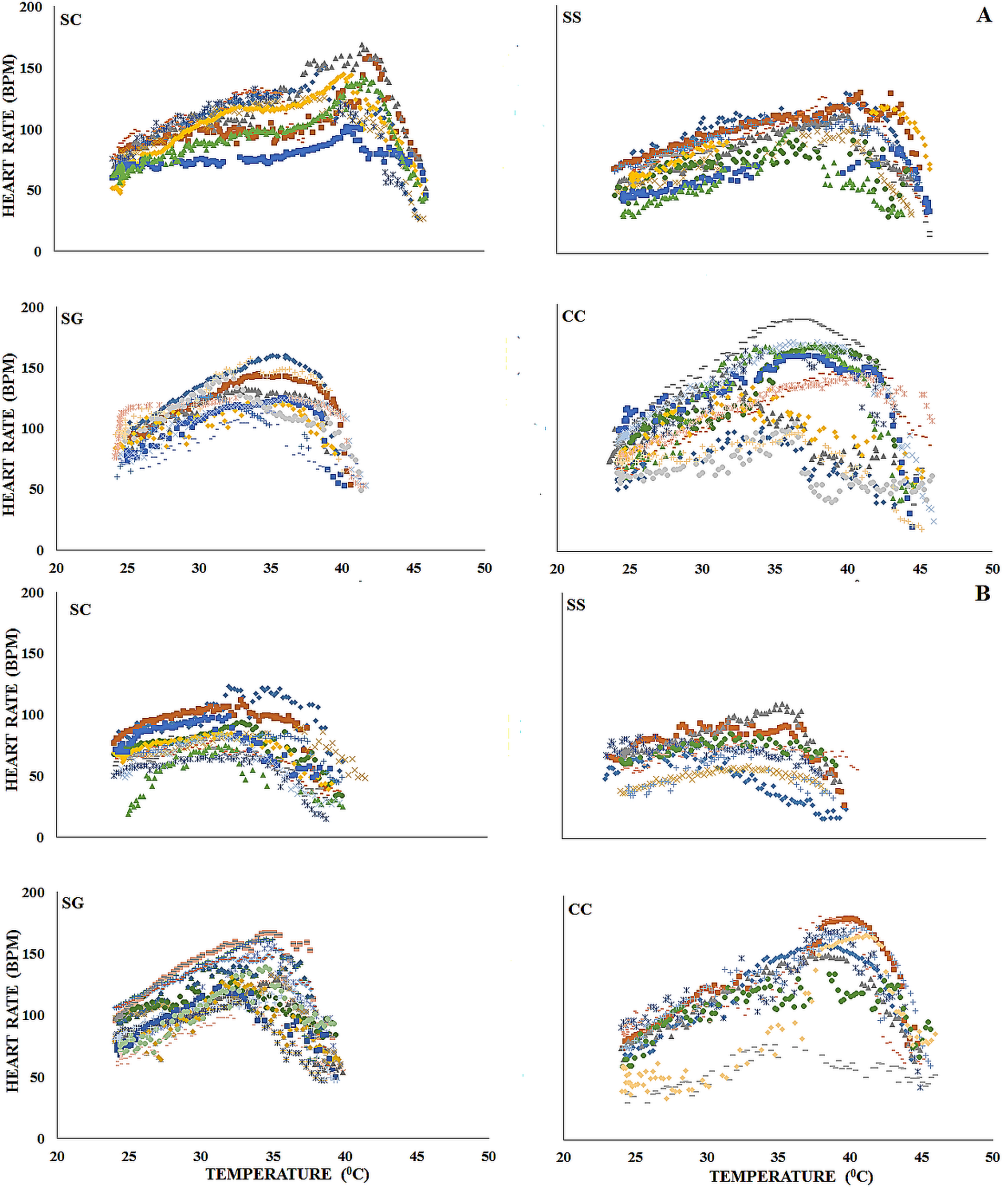
**The cardiac thermal response curves of the study species in air (A) and water (B). SC–*Siphonaria capensis*; SG–*Scutellastra granularis*; SS–*Siphonaria serrata*; CC—*Cellana capensis*.** Each colour represents a different individual.

**Fig 3:**
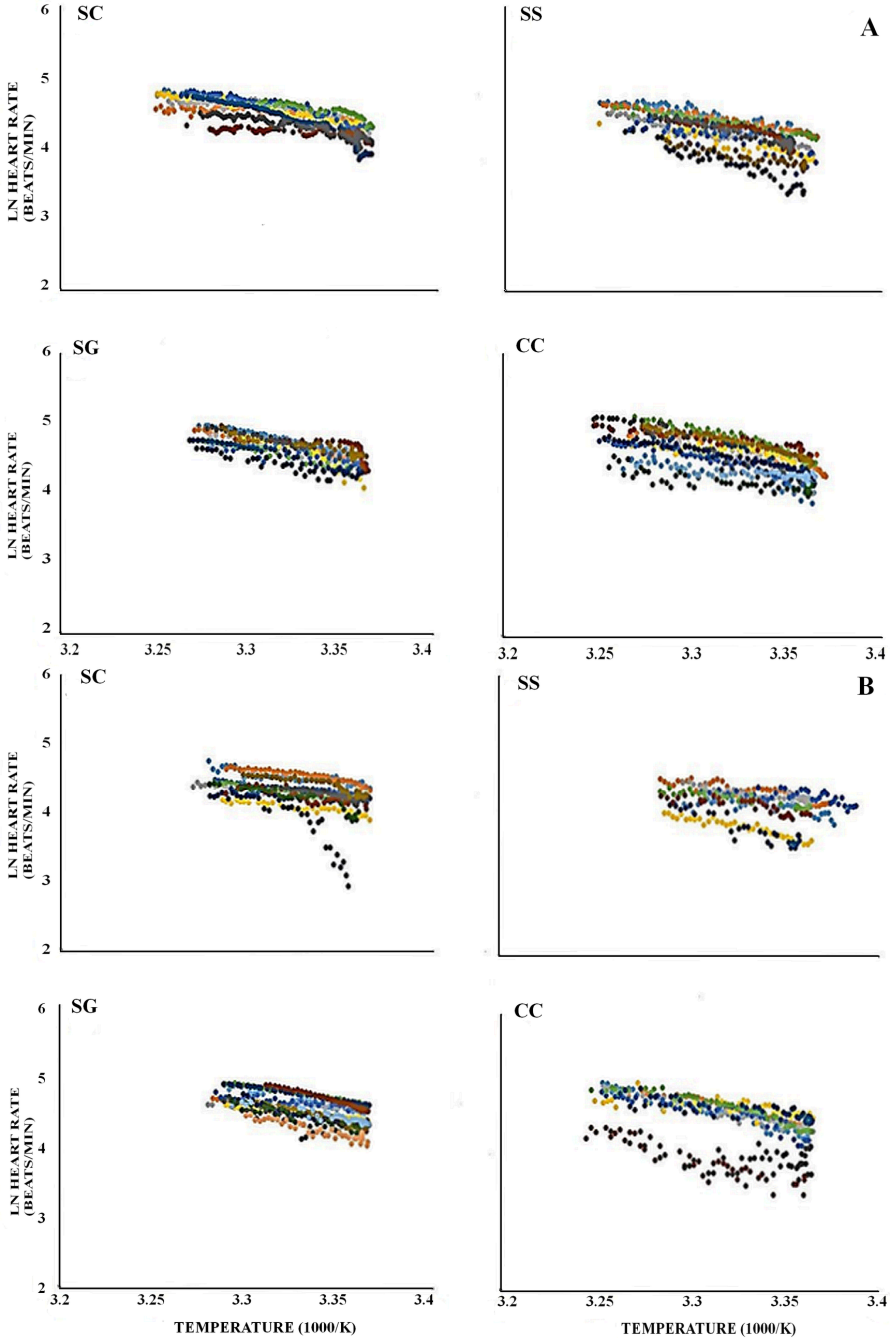
**Arrhenius plots of the study species in water (A) and air (B). SC–*Siphonaria capensis*; SG–*Scutellastra granularis*; SS–*Siphonaria serrata*; CC—*Cellana capensis*.** Each colour represents a different individual.

The cardiac thermal response curves (Fig 2) and Arrhenius plots (Fig 3) displayed a high degree of inter-individual variability within each species in both media.

**Fig 4:**
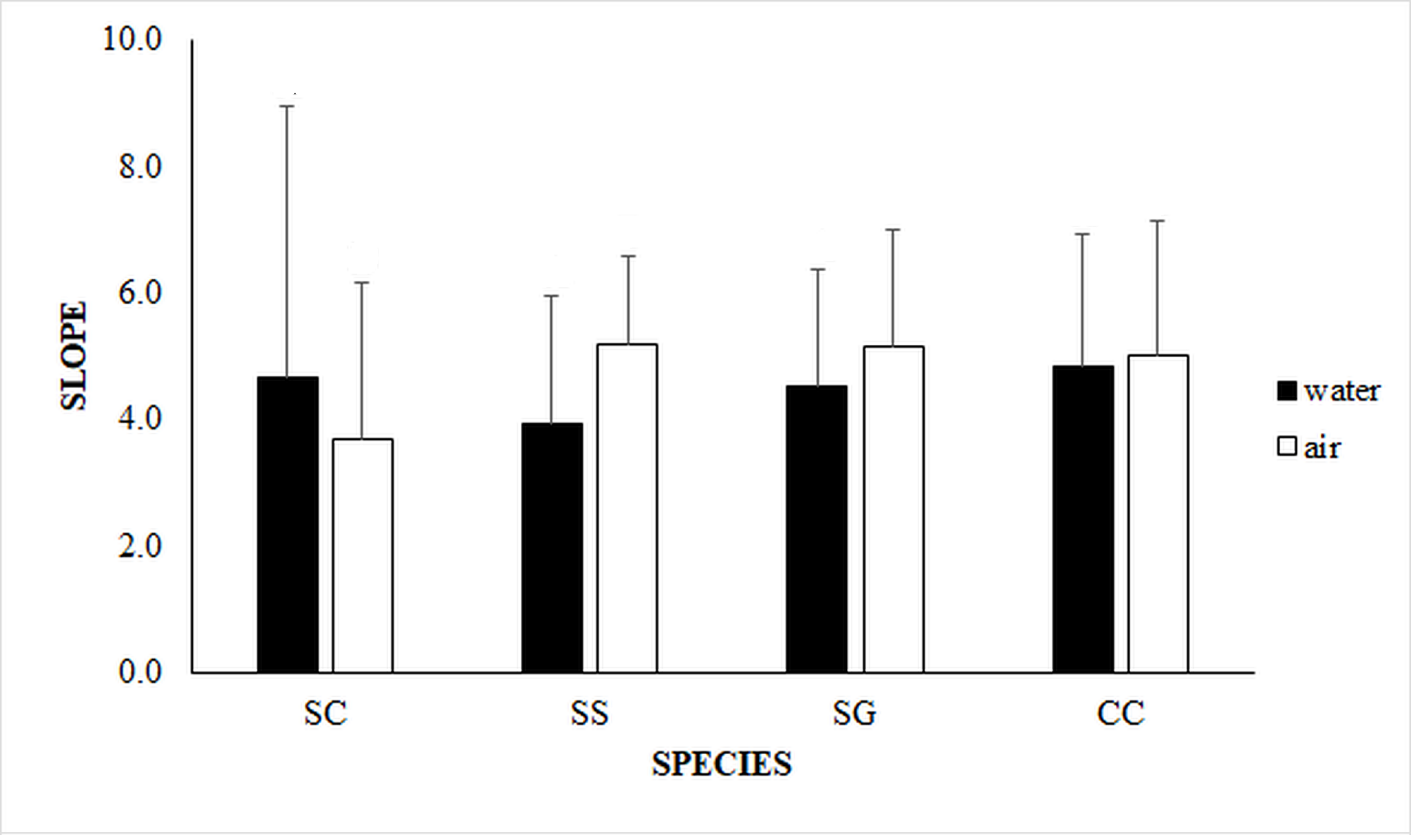
Comparisons of mean (+ S.D) slope values made among the limpet species in both media, and between media for each species. Limpet species and corresponding respiratory mode are listed as: SC–*Siphonaria capensis*, Lungs; SG–*Scutellastra granularis*, Gills; CC–*Cellana capensis*, Gills; SS–*Siphonaria serrata*, Lungs.

The nested ANOVA comparing slopes (S5 Table) revealed insignificant effects for all factors (Fig 4).

## Discussion

We hypothesised that differences in the respiratory morphology of the two limpet groups would be reflected in differences in their responses to increasing temperatures. While the nested analysis for LT_50_ did show a significant effect of respiratory mode, the results from both the LT_50_ and ABT analyses suggested species-specific effects, rather than an over-riding influence of respiratory morphology. A lower aerial temperature tolerance and aquatic LT_50_ was measured for *Scutellastra granularis* compared to the other three species (which did not differ). Similarly, *C*. *capensis* had a higher ABT in water than the other three species whose aquatic ABTs were similar. In addition, there was no obvious effect of medium on species-specific LT_50_ values, while ABT values were generally higher in air except in the case of *C*. *capensis*. There were no significant effects of either respiratory mode or medium on the slopes of the Arrhenius plots.

### Aerial exposure

All experiments performed in air showed important differences between species. *Scutellastra granularis* had the lowest ABT and LT_50_. Conversely, the response of the other patellogastropod was not significantly different to those of the two pulmonates.

The lungs of the Siphonariid limpets were expected to give them a better breathing capability than patellids in air [17, 22, 53, 54, 55], making it easier for them to meet their mitochondrial O_2_ demands. This in turn should allow for the efficient use of energy stores, a delay in the onset of anaerobic metabolism and relatively high upper thermal limits [25, 26, 56, 57].

However, our results indicate that some patellogastropods, like *C*. *capensis*, can survive similar levels of thermal stress to pulmonate limpets during low tide. This may be explained by the fact that some high shore “gill-bearing” limpets can respire efficiently in air despite not having a lung [27, 58–60]. This helps reduce the accumulation of anaerobic by products and water loss, allowing for an increase in aerial thermal tolerance [25, 61, 62]. For example, unexpectedly high thermal limits in air have been measured for the high shore species *Cellana toreuma* (LT_50_ = 41.29–43.36°C) [63] and *C*. *grata* (ABT = 47°C) [35].

### Immersion

Increasing water temperature affected the limpet species differently. The two pulmonates had similar ABT and LT_50_ values, while the two patellogastropods reacted differently. *Scutellastra granularis* had lower thermal limits compared to *Cellana capensis*, which had an exceptionally high ABT.

Pulmonate limpet accessory gills evolved secondarily after loss of the ctenidium, and are not primarily adapted to aquatic respiration, in contrast to the pallial gills of patellogastropod limpets [22, 28]. Despite this, the pulmonate limpets had surprisingly high thermal limits in water, probably due to the efficient use of their accessory gills as shown previously by Koopman *et al*. [64]. These authors found that, when submerged the freshwater pulmonate limpets *Physa fontinalis* and *P*. *acuta* had higher upper thermal limits (CT_max_) than the gill-bearing caenogastropods *Bithynia tentaculata* and *Potamopyrgus antipodarum*.

### Air vs water

When comparing thermal performance in air and water, there were strong similarities between the two limpet groups. For both groups, there was no significant effect of medium when performance was measured as LT_50_. In the case of the pulmonates, both species showed significantly higher ABT values in air. Among the patellogastropods, the same was true for *Scutellastra granularis*, but not *C*. *capensis*. Regarding the slopes from the Arrhenius plots, the non-significant effects were probably related to the high inter-individual variability (Fig 4) in the species sensitivity to increasing temperature. The slope of the heart rate response represents thermal metabolic sensitivity [17, 35, 37, 55, 65], and this has previously been shown to be highly variable inter-individually [66].

Past studies have generally focused on the influence of geographic [37, 67] or vertical distribution [35, 36, 68] on the thermal tolerances of intertidal organisms. Most such studies examined thermal tolerance in submersed animals, with only a few examining aerial thermal tolerances or comparing tolerances in different media [36, 62, 69]. Even fewer studies have compared the aerial thermal limits of air and water breathing gastropod molluscs [but see 26, 70, 71].

Aquatic animals adapted to aerial respiration (such as pulmonate limpets) should be able to respire more efficiently in air where oxygen concentrations are higher, but the emersion period is also characterized by extreme temperature and desiccation stress involving important energetic costs [25, 72–75]. Dye [76] found that *Siphonaria capensis* and *S*. *concinna* had higher respiration rates in air than water, indicating that it would be easier to avoid anaerobic metabolism, which should translate into higher aerial thermal limits [77–79].

Because patellogastropods respire primarily through their pallial gills, they were expected to have lower thermal limits in air. In the case of LT_50_, there was no difference between media for either *Scutellastra granularis* or *Cellana capensis*, while the ABT for *S*. *granularis* was unexpectedly higher in air. This has also been shown for the mid-to-high shore patellogastropod limpet *Lottia digitalis*, which had a higher thermal limit (final cardiac breakpoint temperature) in air [80], and in both cases, this presumably reflects the efficiency of O_2_ uptake by the highly vascularised mantle cavity of many high shore patellogastropod limpets.

### Conclusions

Although the results differed slightly between LT_50_ and ABT, they provide no clear indication that respiratory morphology is important in determining either aerial or aquatic thermal limits in the study species, indicating that other factors play important roles. Within species, reproductive and nutritional state can influence susceptibility to high temperatures, and probably contributed to the high degree of individual variability we observed in the relationship between the limpet heart rates and temperature [81–85]. Recent thermal history, including the influence of microhabitat use, is also likely to influence thermal limits [16, 30, 74, 86–88]. In this regard, body temperature estimates generated from the range of microhabitats occupied by each species would have benefited this study. Nevertheless, our overall conclusion is that the data do not support the hypothesis that the respiratory morphology of these species has an overriding influence on the interaction of thermal tolerance and respiratory medium.

## Supporting information

S1 Fig Map displaying the study animals’ distributions along the South African coast line. SC–*Siphonaria capensis*; SG–*Scutellastra granularis*; SS–*Siphonaria serrata*; CC—*Cellana capensis*.
(JPG)

S2 Fig Graphic (not to scale) displaying the vertical zonation (m) patterns (approximates) of the study animals. Vertical zonation patterns were derived from Allanson [38], Branch [39, 41] and, Chambers and McQuaid [42]. Limpet species and intertidal zones are listed as: SC–*Siphonaria capensis*; SG–*Scutellastra granularis*; SS–*Siphonaria serrata*; CC—*Cellana capensis*. LZ–Littorina Zone; HMZ–High Mid-Shore Zone; LMZ–Low Mid-Shore Zone; SFZ–Subtidal Fringe Zone.
(PNG)

S3 Fig Graphic (not to scale) of the LT_50_ ramping protocol for the three highest temperature intervals used in this study. Mortality (nr of individuals) was determined at the 5 and 29 hour marks.
(TIF)

S1 Table The temperature intervals and in parentheses the heating rates (°C.min^-1^) used to determine the limpets LT_50_ values in both media. (DOCX)

S2 Table The final number of replicates per species for each experimental condition. SC–*Siphonaria capensis*; SG–*Scutellastra granularis*; SS–*Siphonaria serrata*; CC—*Cellana capensis*.
(DOCX)

S3 Table Results from the nested design ANOVA run on the LT_50_ (°C) values obtained via probit analysis of the limpet’s mortality (%) vs temperature (°C) data in both media. Medium, Respiration mode (R. Mode) and Species nested in Respiration mode (R. Mode) were considered as fixed factors.
(DOCX)

S4 Table Results from the nested design ANOVA run on the ABTs obtained from the thermal response curves in both media. Medium, Respiration mode (R. Mode) and Species nested in Respiration mode (R. Mode) were considered as fixed factors.
(DOCX)

S5 Table Results from the nested design ANOVA run on the slopes obtained from the thermal response curves in both media. Medium, Respiration mode (R. Mode) and Species nested in Respiration mode (R. Mode) were considered as fixed factors.
(DOCX)
