# Supplementary figures and images for: Influence of respiratory mode on the thermal tolerance of intertidal limpets
Source: PLoS One. 2018 Sep 5;13(9):e0203555. doi: 10.1371/journal.pone.0203555 (PMC6124786; doi:10.1371/journal.pone.0203555)

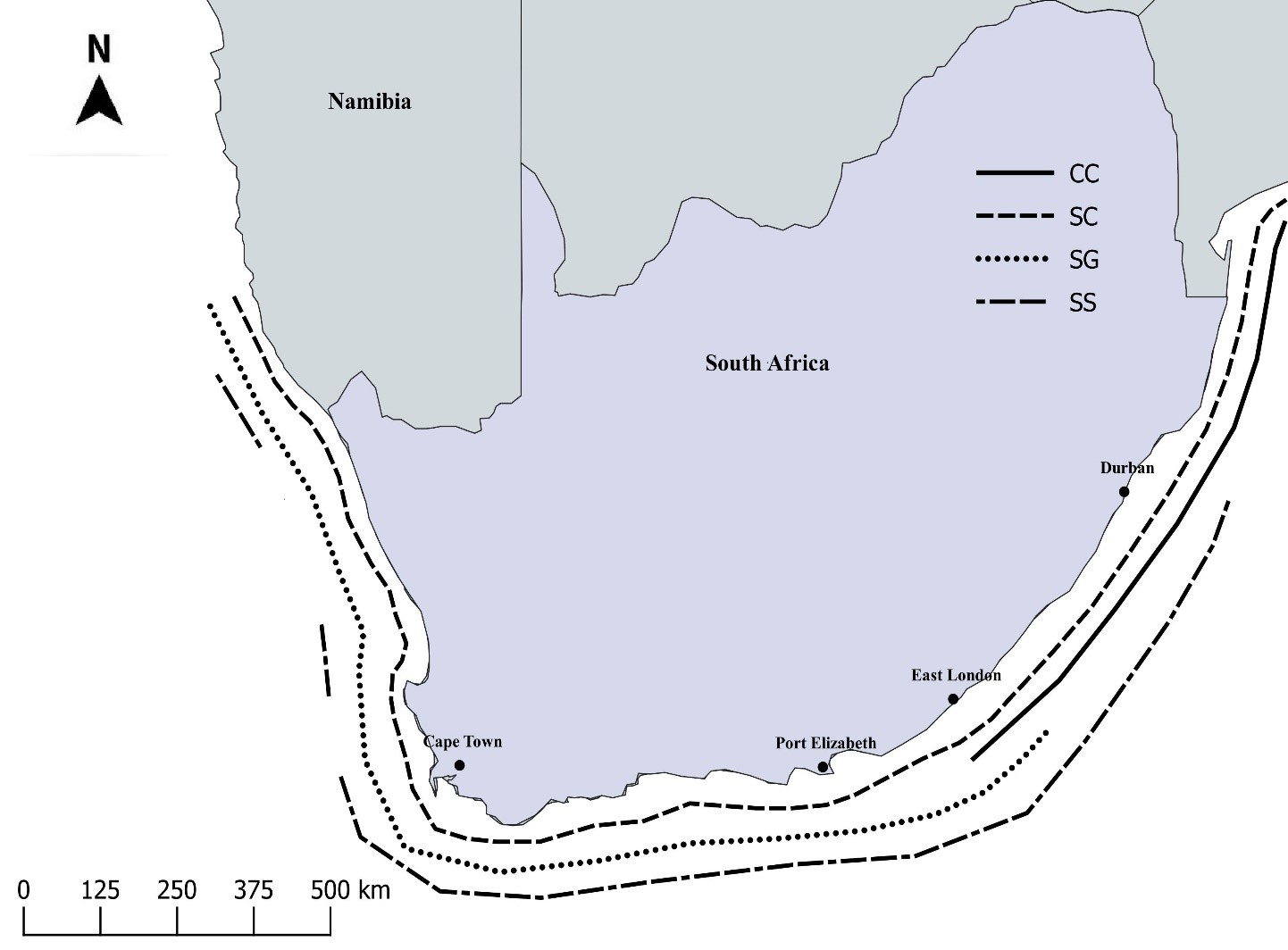

Supplement: S1 Fig — SC–Siphonaria capensis; SG–Scutellastra granularis; SS–Siphonaria serrata; CC—Cellana capensis. (JPG) [file pone.0203555.s001.jpg]

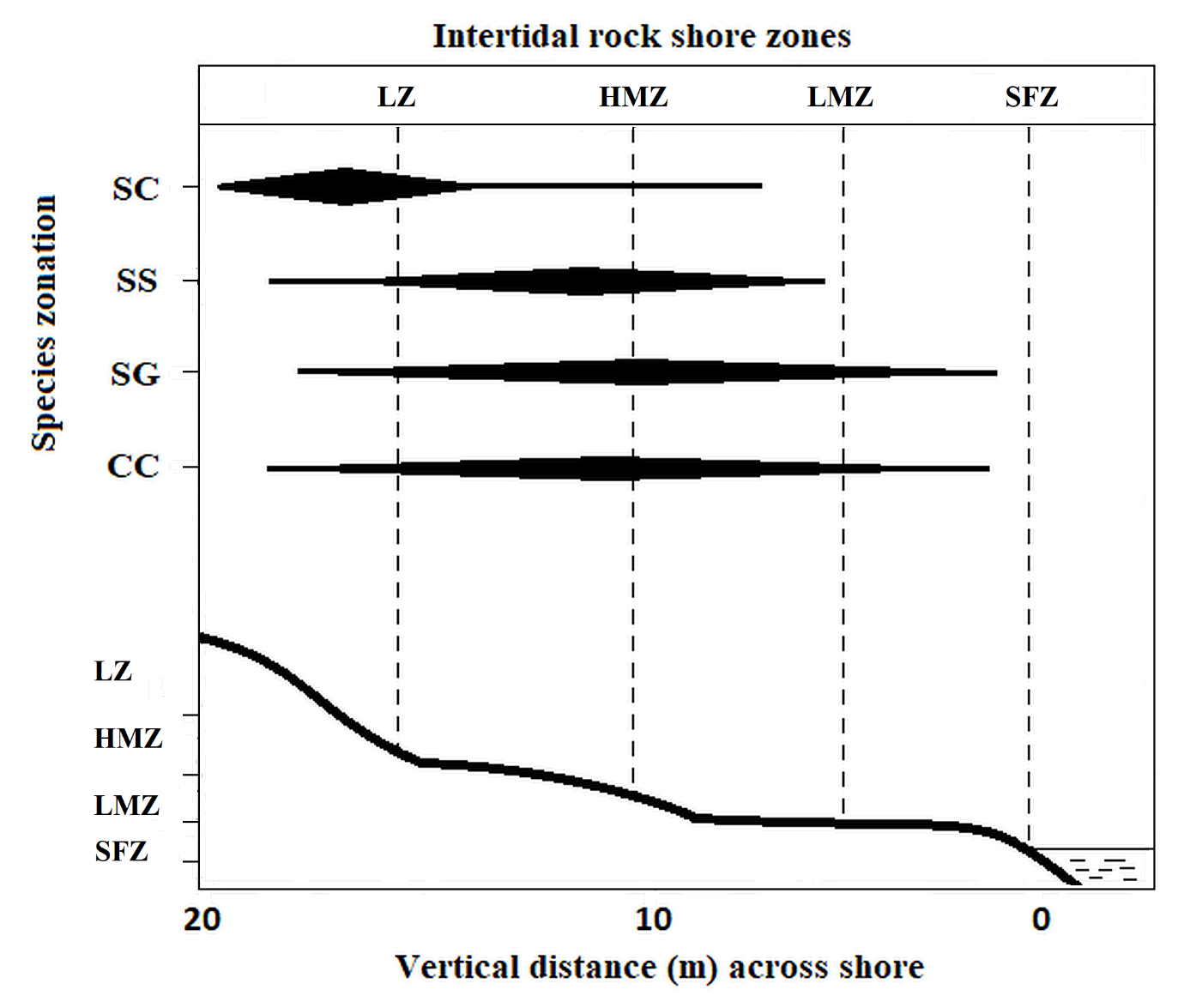

Supplement: S2 Fig — Vertical zonation patterns were derived from Allanson [38], Branch [39, 41] and, Chambers and McQuaid [42]. Limpet species and intertidal zones are listed as: SC–Siphonaria capensis; SG–Scutellastra granularis; SS–Siphonaria serrata; CC—Cellana capensis. LZ–Littorina Zone; HMZ–High Mid-Shore Zone; LMZ–Low Mid-Shore Zone; SFZ–Subtidal Fringe Zone. (PNG) [file pone.0203555.s002.png]

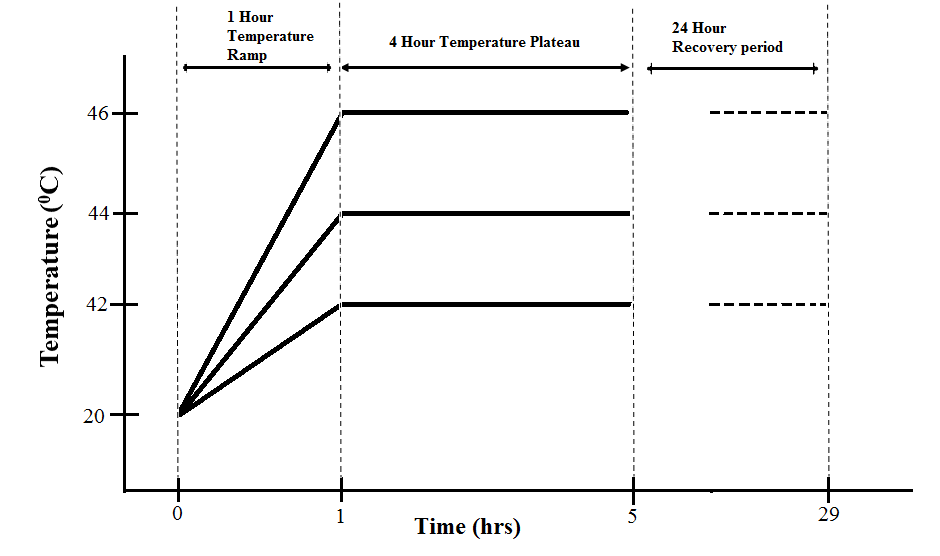

Supplement: S3 Fig — Mortality (nr of individuals) was determined at the 5 and 29 hour marks. (TIF) [file pone.0203555.s003.tif]
