# Supplementary material for: Influence of respiratory mode on the thermal tolerance of intertidal limpets
Source: PLoS One. 2018 Sep 5;13(9):e0203555. doi: 10.1371/journal.pone.0203555 (PMC6124786; doi:10.1371/journal.pone.0203555)
Supplement: S1 Table — (DOCX) [file pone.0203555.s004.docx]

|  | **LT_50_ Temperature Intervals (^°^C)** | | | | | | | | | |
| --- | --- | --- | --- | --- | --- | --- | --- | --- | --- | --- |
| **Species** | **Medium** | | | | | | | | | |
|  | **Air** | | | | | **Water** | | | | |
| ***Siphonaria capensis*** | 38 (0.3) | 40  (0.33) | 42  (0.36) | 44 (0.4) | 46  (0.43) | 38 (0.3) | 40  (0.33) | 42  (0.36) | 44 (0.4) | 46  (0.43) |
| ***Scutellastra granularis*** | 34  (0.23) | 36  (0.26) | 38 (0.3) | 40  (0.33) | 42  (0.36) | 34  (0.23) | 36  (0.26) | 38 (0.3) | 40  (0.33) | 42  (0.36) |
| ***Siphonaria serrata*** | 38 (0.3) | 40  (0.33) | 42  (0.36) | 44 (0.4) | 46  (0.43) | 38 (0.3) | 40  (0.33) | 42  (0.36) | 44 (0.4) | 46  (0.43) |
| ***Cellana capensis*** | 38 (0.3) | 40  (0.33) | 42  (0.36) | 44 (0.4) | 46  (0.43) | 38 (0.3) | 40  (0.33) | 42  (0.36) | 44 (0.4) | 46  (0.44) |
