# Supplementary material for: Influence of respiratory mode on the thermal tolerance of intertidal limpets
Source: PLoS One. 2018 Sep 5;13(9):e0203555. doi: 10.1371/journal.pone.0203555 (PMC6124786; doi:10.1371/journal.pone.0203555)
Supplement: S2 Table — SC–Siphonaria capensis; SG–Scutellastra granularis; SS–Siphonaria serrata; CC—Cellana capensis. (DOCX) [file pone.0203555.s005.docx]

| **Species** | **Medium** | |  |
| --- | --- | --- | --- |
|  | **Air** |  | **Water** |
| **SC** | 9 |  | 12 |
| **SG** | 11 |  | 14 |
| **SS** | 11 |  | 8 |
| **CC** | 13 |  | 9 |
