# Supplementary material for: Influence of respiratory mode on the thermal tolerance of intertidal limpets
Source: PLoS One. 2018 Sep 5;13(9):e0203555. doi: 10.1371/journal.pone.0203555 (PMC6124786; doi:10.1371/journal.pone.0203555)
Supplement: S3 Table — Medium, Respiration mode (R. Mode) and Species nested in Respiration mode (R. Mode) were considered as fixed factors. (DOCX) [file pone.0203555.s006.docx]

| **ANOVA**  **Factors** | **LT_50_ (^°^C)** | | | |
| --- | --- | --- | --- | --- |
|  | **MS** | **df** | **F** | **p** |
| **Medium** | 0.8 | 1 | 0.4 | p=0.55 |
| **R. Mode** | 47.06 | 1 | 218.5 | p<0.0001 |
| **Spp (R. Mode)** | 39.74 | 2 | 184.5 | p<0.0001 |
| **Medium*R.**  **Mode** | 0.34 | 1 | 1.6 | p=0.23 |
| **Error** | 18 | 0.22 |  |  |
