# Supplementary material for: Influence of respiratory mode on the thermal tolerance of intertidal limpets
Source: PLoS One. 2018 Sep 5;13(9):e0203555. doi: 10.1371/journal.pone.0203555 (PMC6124786; doi:10.1371/journal.pone.0203555)
Supplement: S5 Table — Medium, Respiration mode (R. Mode) and Species nested in Respiration mode (R. Mode) were considered as fixed factors. (DOCX) [file pone.0203555.s008.docx]

| **ANOVA**  **Factors** | **SLOPES (^°^C)** | | | |
| --- | --- | --- | --- | --- |
|  | **MS** | **df** | **F** | **p** |
| **Medium** | 1.44 | 1 | 0.24 | p=0.62 |
| **R. Mode** | 3.76 | 1 | 0.64 | p=0.43 |
| **Spp (R. Mode)** | 0.85 | 2 | 0.14 | p=0.87 |
| **Medium*R.**  **Mode** | 0.66 | 1 | 0.11 | p=0.74 |
| **Error** | 5.91 | 81 |  |  |
